# Cell Size Modulates Oscillation, Positioning and Length of Mitotic Spindles

**DOI:** 10.1038/srep10504

**Published:** 2015-05-27

**Authors:** Hongyuan Jiang

**Affiliations:** 1 Department of Modern Mechanics, CAS Key Laboratory of Mechanical Behavior and Design of Materials, University of Science and Technology of China, Hefei, Anhui 230026, China

## Abstract

Mitotic spindle is the main subcellular structure that accomplishes the chromosome segregation between daughter cells during cell division. However, how mitotic spindles sense and control their size, position and movement inside the cell still remains unclear. In this paper, we focus on the size effects of mitotic spindles, i.e., how cell size controls various interesting phenomena in the metaphase, such as oscillation, positioning and size limit of mitotic spindles. We systematically studied the frequency doubling phenomenon during chromosome oscillation and found that cell size can regulate the period and amplitude of chromosome oscillation. We found that the relaxation time of the positioning process increases exponentially with cell size. We also showed that the stabler microtubule-kinetochore attachments alone can directly lead to an upper limit of spindle size. Our work not only explains the existing experimental observations, but also provides some interesting predictions that can be verified or rejected by further experiments.

Mitotic spindle, a bipolar assembly of dynamic microtubules and various proteins, is the main subcellular structure that accomplishes the chromosome segregation between daughter cells during cell division. For a long period of time, the spindle size was expected to be proportional to the cell size since the cell size is the main control parameter determining the size of subcellular structures^1^,^2^,^3^. However, recent experiments found that the size of the mitotic spindle scales with the cell size only in small cells, but approaches an upper limit (about 60 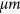) in large cells during the early embryogenesis of Xenopus laevi eggs^4^. Similar phenomena can be recapitulated by *in vitro* experiments^5^,^6^, where spindles containing kinetochores and centrosomes are assembled in Xenopus egg extracts. Although it has been shown that the spindle size can be tuned by certain factors, such as the number of building blocks^5^,^6^, the morphogen gradient^7^, or the growth velocity of microtubules^8^, how mitotic spindles sense the cell size and accordingly regulate their own size still remains unclear.

Cell size is also expected to be an important control parameter for some interesting phenomena, such as the positioning and oscillation of spindle and chromosome. Correct and accurate positioning of the mitotic spindle plays an important role in chromosome segregation and selection of the cell division plane^9^,^10^. Either the pushing forces generated by the polymerization of microtubules^11^,^12^,^13^,^14^ or the pulling forces generated by the walking of molecular motors^15^,^16^,^17^ can position microtubule organizing centers (MTOC) or spindles to the geometry center of the cell. The combination of pulling and pushing forces provides a more robust mechanism for the spindle positioning^18^,^19^,^20^. Chromosomes positioned near the metaphase plate can undergo directional instability, i.e., sustained chromosome oscillations between the two spindle poles during the metaphase of cell division^21^. Positioning the spindle inside the cell and driving the sustained chromosome oscillation require the sensing and control on the position and movement of chromosome through length-dependent or position-dependent forces^1^. Therefore, cell size should be a key control parameter for these processes. Although various models^22^,^23^,^24^,^25^,^26^,^27^,^28^,^29^ have been developed, how cell size regulates the positioning and oscillation of spindle and chromosome is still unknown.

Furthermore, although the previous models^22^,^23^,^24^,^25^,^26^,^27^,^28^,^29^ can reproduce the chromosome oscillation and positioning phenomenon qualitatively, they cannot explain the fine behaviors of the mitotic spindle discovered by some recent experiments. For example, recently it has been shown that the transitions from the poleward (P) to the away-from-the-pole (AP) movement of the chromosomes, i.e., P-to-AP reversals, always occur 6 seconds before AP-to-P reversals^30^ and chromosomes oscillate at a period twice that of the oscillation of inter-kinetochore distance^30^,^31^,^32^. Previous studies usually treat the oscillation and positioning of mitotic spindles as isolated problems and investigate them separately. For example, in most models about chromosome oscillation^22^,^23^,^24^,^25^,^26^,^27^,^28^,^29^, astral microtubules and cell boundary are neglected so that the positions of the spindle poles and the spindle size are fixed. Therefore, these models cannot be used to investigate the positioning of the whole spindle structure and how cell size regulates the oscillation and positioning. In contrast, in the previous models about the positioning process^12^,^14^,^18^,^19^,^20^, the whole complex spindle structure is usually represented by a single point, i.e., one MTOC. Therefore, these simplified models cannot be used to study the chromosome oscillation and how the spindle size is determined by the cell size. Therefore, in order to study all these interesting phenomena in a single model and find the most essential factors regulating these phenomena, a minimal but general model with as few parameters as possible should be developed.

In this paper, we will focus on the size effects of mitotic spindles, i.e., how cell size regulates the oscillation, positioning and size limit of mitotic spindles. Here we will show that the oscillation, positioning and size limit of mitotic spindles can be studied in a general model by considering the properties that are intrinsic to the spindle, such as the growth dynamics of microtubules, the pulling forces generated by molecular motors, the pushing forces limited by the buckling force or stall force of microtubules, and the difference between microtubule-kinetochore attachments and microtubule-cortex attachments. We will show that cell size can regulate the period and amplitude of chromosome oscillation. We’ll also show that the relaxation time of the positioning process increases exponentially with cell size. Finally, we will demonstrate that the stabler attachments between microtubule and kinetochore can directly lead to an upper limit of spindle size.

## Results

### A minimal model to study the oscillation, positioning and size limit of mitotic spindles

**Figure 1:**
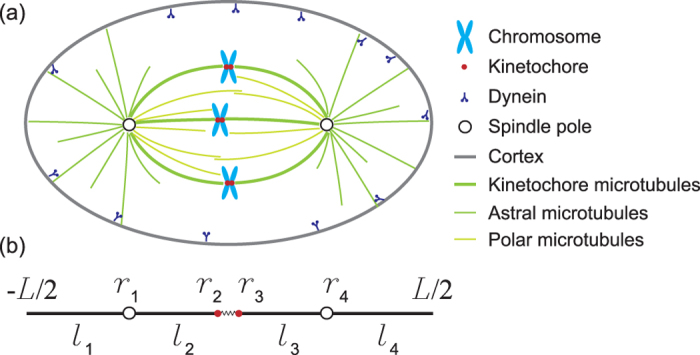
(**a**) Schematic showing a mitotic spindle inside a cell. (**b**) A simplified 1D force balance model. Two poles (*r*_1_ and *r*_4_) and one pair of sister chromatids (*r*_2_ and *r*_3_) are connected by aMTs and kMTs with length *l*_1_, *l*_2_, *l*_3_ and *l*_4_. The position and the size of the spindle are given by (*r*_1_ + *r*_4_)/2 and *r*_4_ − *r*_1_, respectively. The two sister chromatids is simply connected by a linear spring with rest length Δ and spring constant *α*.

As shown in Fig. 1a, the mitotic spindle has two poles and each pair of sister chromatids has two attachment sites, named kinetochores. Microtubules are nucleated from the spindle poles and undergo rapid stochastic switching between growth and shrink states (dynamic instability of microtubules) to search for kinetochores^33^,^34^. Once both kinetochores of the sister chromatids are caught by microtubules, tension are built up and stabilize the whole structure. In the meanwhile, the sister chromatids are still mechanically connected by cohesin protein complexes until anaphase. Therefore, the duplicated chromosomes are aligned near the spindle equator to form the metaphase plate during the metaphase.

In this paper, we consider a one-dimensional (1D) cell with size 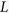 (Fig. 1b). Inside the cell, there are two poles at 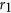 and 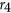, and one pair of sister chromatids at 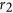 and 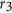. The spindle size can be characterized by the distance between the two spindle poles, i.e., 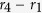. The position of the spindle, i.e., the center of the spindle, is given by 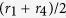. Microtubules can grow from the two poles and catch chromatids or reach the cell periphery. 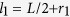 and 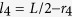 represent the length of astral microtubules (aMTs), while 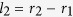 and 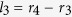 indicate the length of kinetochore-associated microtubules (kMTs). Notice that the spatial and temporal regulation of microtubules and associated proteins inside the spindle is quite complex and motors are almost everywhere inside the spindle. To reduce this complexity, we follow the previous models about spindle structure and chromosome oscillation to assume that microtubules are directly connected to kinetochores^22^,^23^,^24^,^25^,^28^. The mechanical connection between the two sister chromatids is simplified to be a linear spring with rest length 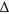 and spring constant 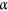. Following Ref. [14,19,20], we consider two populations of microtubules in the 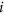-th microtubule segment (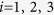 or 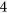): pushing microtubules with number 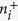 and pulling microtubules with number 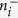, where + and 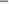 indicate the pushing and pulling, respectively. The force balance equations on the poles are


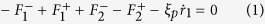



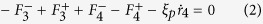


and the force balance equations on the chromatids are


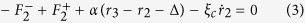



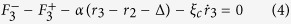


where *ξ*_*p*_ and *ξ*_*c*_ are the viscous drag coefficients of spindle poles and chromatids, respectively. 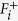 and 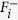 are the pushing and pulling forces in the *i*-th microtubule segment with length *l*_*i*_ (Fig. 1b). Here we have neglected inertial forces since Reynolds number is very low in this system. We also neglected the viscous forces on the microtubules because they are negligible compared to other forces considered here^35^.

When the plus end of a microtubule growing from a spindle pole makes contact with some object, it will apply a pushing force on the object. If the pushing force exceeds a critical value, the growing microtubule will be buckled due to its own compression. Experiments have shown that high percentage of free microtubules will buckle when they reach the cell cortex or barrier^20^,^36^,^37^. Therefore, we assume that the pushing force 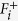 is limited by this critical force^14^,^19^,^20^, which is given by Euler buckling formula *f*_*c*_ = *π*^2^*κ*/*l*^2^. Here *κ* and *l* denote the bending rigidity and the length of microtubules, respectively. By solving the post-buckling shape of the microtubule, one can show that the pushing force increases very slowly with the growth of microtubule after it exceeds the Euler buckling force. Therefore, given that the catastrophe rate is high, the deflection of the buckled microtubule will be small and we can assume the pushing force is approximately equal to the Euler buckling force^14^,^19^,^20^. Notice that when microtubules are very short, the Euler buckling force *f*_*c*_ will exceed the stall force of microtubule *F*_*s*_. So the pushing force generated by the polymerization of microtubules can be given as


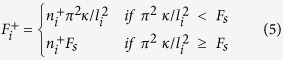


The lateral reinforcement by the surrounding elastic cytoskeleton could greatly increase the critical force^38^. In this case, the critical force is *f*_*c*_ = 8*π*^2^*κ*/*b*^2^, where *b* is the buckling wavelength instead of the microtubule length. Considering this effect only changes our results quantitatively. Notice that the pushing force given in equation (5) is naturally position-dependent without any other assumptions. Therefore, buckled microtubules are not only force generators, but also the simplest rulers to sense the position inside the cell.

Some molecular motors, such as dyneins, can walk to microtubule minus ends while bound to the cell cortex or kinetochore so that they can generate pulling forces. In contrast, kinesins usually walk to microtubule plus ends and therefore generate a pushing force. The force generated by motors is 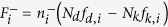, where *N*_*d*_ and *N*_*k*_ are the number of dyneins and kinesins on each microtubule, respectively. *f*_*d*,*i*_ and *f*_*k*,*i*_ are the force generated by each dynein and kinesin. The velocity of dyneins and kinesins has been shown to be strongly influenced by the load^39^,^40^,^41^,^42^. The velocity and force of dyneins can be modeled as *v*_*d*,*i*_ = *v*_*d*,0_(1 − *f*_*d*,*i*_/*f*_*d*,*s*_), where *f*_*d*,*s*_ and *v*_*d*,0_ are the stall force and unloaded velocity of the dynein, *f*_*d*,*i*_ is the pulling force on the dynein. Similarly, the velocity and force of kinesins can be written as *v*_*k*,*i*_ = *v*_*k*,0_(1 − *f*_*k*,*i*_/*f*_*k*,*s*_). Therefore, by introducing *λ* = *N*_*d*_/(*N*_*d*_ + *N*_*k*_) and *N* = *N*_*d*_ + *N*_*k*_, the force generated by motors can be given as


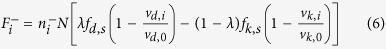


If *λ* = 1, only dyneins apply forces on the microtubule. In contrast, if *λ* = 0, only kinesins generate forces. In most cases, dyneins dominate the force generation so that the resultant force generated by motors is a pulling force^25^. The walking velocities of motors relative to microtubules are related to the motion of spindle poles and chromatids by 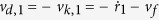, 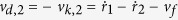, 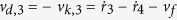 and 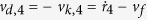, where *v*_*f*_ is the poleward flux of microtubules.

It should be noted that the effects of polar ejection forces have been carefully considered in this model. Polar ejection forces can be generated by the pushing of polymerizing microtubule plus ends against the chromosomes or by chromokinesin motors^1^. The polar ejection force due to the polymerization of microtubules has been given by 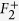 and 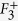. And the polar ejection force generated by kinesins has been included in equation (6). Notice that polar ejection forces were proposed to decrease with the distance from the spindle pole^23^ and the assumption has been verified experimentally^43^. This is consistent with the formula of 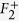 and 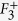. It should also be noted that although the inhibition of chromokinesin motors reduces chromosome-to-pole distance in monopoles^1^, kinetochores are on average under tension^44^. This indicates that pulling force applied by dyneins is bigger than the pushing force generated by kinesins in equation (6) so that 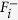 should be a pulling force. Therefore, without loss of generality, we can assume *λ* = 1 in our simulation, i.e., only dyneins contribute to the force generation in equation (6). In this case, equation (6) is reduced to


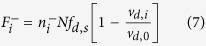


Finally, we assume that the pushing forces are exerted on the chromosome arms, while the pulling forces are exerted on the kinetochore. In another word, we assume that kMTs only provide pulling forces in our model and we don’t have to consider the buckling of kMTs. When we simplify the model to 1D case, the chromosome arm and kinetochore are reduced to a single point (*r*_2_ or *r*_3_) as shown in Fig. 1b. Therefore, by using the 1D approximation, we cannot distinguish the different locations of the chromosome arms and kientochores. This is one major limitation of this 1D model. If we extend this model to 2D and 3D cells to study the orientation of the spindle, we must consider the population evolution of microtubules connected to the chromosome arms and kinetochores separately.

The two populations of microtubules 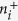 and 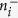 are mainly determined by the binding and unbinding rate of motors, the catastrophe rate of microtubules, and the number of microtubules reaching the cell cortex or chromosome per unit time^14^,^19^. Therefore, the time evolution of the two populations of microtubules can be described by the following equations


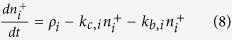



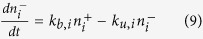


where *ρ*_*i*_ is the number of microtubules reaching the cell cortex or chromosome per unit time, *k*_*c*,*i*_ is the catastrophe rate of pushing microtubules, *k*_*b*,*i*_ is binding rate of motors to pulling microtubules, and *k*_*u*,*i*_ is unbinding rate of motors from pulling microtubules. The unbinding rate *k*_*u*,*i*_ increases exponentially with the applied load as 
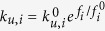
 Ref. [22,25], where 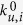 is the unloaded unbinding rate, 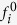 is characteristic force indicating the sensitivity of the unbinding rate to the applied load. It should be noted that following the previous models about chromosome oscillation^22^,^23^,^24^,^25^,^28^, we assume that microtubules are directly connected to kinetochores and only motors binding to or unbinding from the two ends of microtubule contribute to evolution of the two populations of microtubules. Notice that the dynamic instability of microtubules can affect the value of *ρ*_*i*_ and in general *ρ*_*i*_ should be the function of the distance between the spindle pole and the destination. How the dynamic instability of microtubules influences the formula of *ρ*_*i*_ will be discussed in the last section.

### Frequency doubling and regulation of chromosome oscillation by cell size

**Figure 2:**
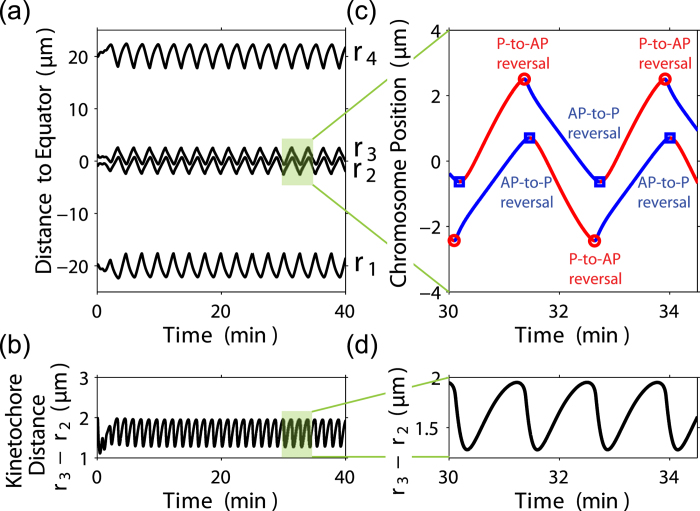
Frequency doubling during directional instability. (**a**) The oscillation of chromosomes (*r*_2_ and *r*_3_) and spindle poles (*r*_1_ and *r*_4_). (**b**) The oscillation of inter-kinetochore distance *r*_3_ − *r*_2_. (**c**) and (**d**) show the details of (**a**) and (**b**). Red and blue lines indicate P and AP movement, respectively. Parameters used in the simulation are summarized in the Table 1.

By solving 12 coupled equations, i.e., equation (1)-(4, 22, 23, 24),(8) and (9), we can obtain 12 unknowns. Therefore, we can study the size and position of the spindle (*r*_4_ − *r*_1_ and (*r*_1_ + *r*_4_)/2), and the inter-kinetochore distance (*r*_3_ − *r*_2_). If the fixed point of the above nonlinear equations is stable, chromosomes and spindle poles will approach a steady position. In contrast, if it’s unstable, both chromosomes and spindle poles will undergo sustained periodic oscillations (Fig. 2a) and the oscillation period is about several minutes as observed in experiments^30^,^31^,^45^. Interestingly, chromosomes oscillate at a period twice that of the oscillation of inter-kinetochore distance (Fig. 2a,b), which is consistent with the accurate experimental measurement of the positions of the sister kinetochores^30^,^31^,^32^. The asymmetry between the poleward (P) and away-from-the-pole (AP) moving speed leads to frequency doubling of inter-kinetochore distance (Fig. 2c,d) as proposed in Ref. [31]. We found the P-to-AP reversals occur 6 seconds before AP-to-P reversals in Fig. 2c which is quantitatively consistent with the PtK2 oscillation *in vivo*^30^. Interestingly, spindle poles also oscillate and lag behind the chromosome oscillation by about 180 degrees (Fig. 1a). Notice that we only consider one pair of chromosomes, and therefore we predict the same oscillations period for chromosome and spindle poles. However, their oscillation period and amplitude could be very different due to the coupling dynamics of multiple chromosomes and spindle poles. Also notice that the chromosome oscillation is faster than the oscillation of the whole spindle (the period is about 30 minutes)^46^ since the drag coefficient of the whole spindle structure is much larger than that of a single chromosome.

**Figure 3:**
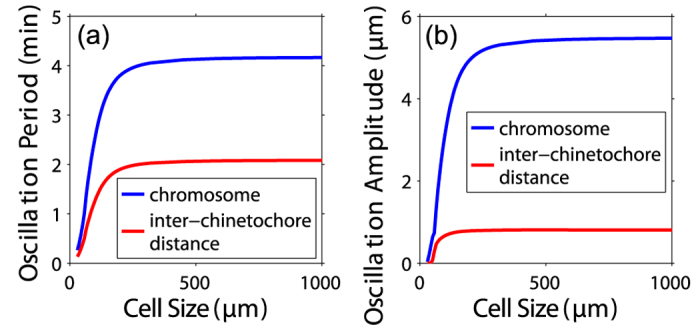
The oscillation period (**a**) and amplitude (**b**) of chromosomes (blue) and inter-kinetochore distance (red) as the function of cell size.

**Figure 4:**
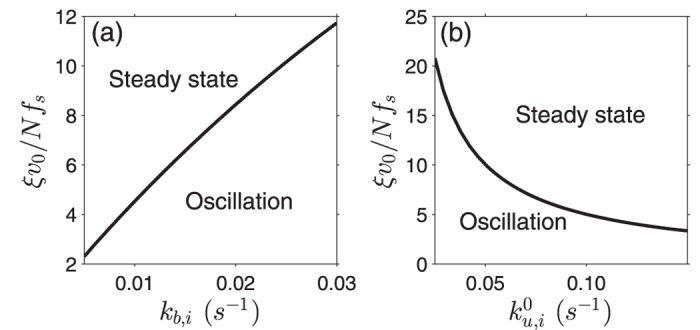
Phase diagram of chromosome movement characterized by dimensionless parameter *ξv*_0_/*Nf*_*s*_ and (**a**) the binding rate *k*_*b*,*i*_ or (**b**) the unbinding rate 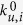 (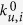 are the same for all *i* in this calculation).

Remarkably, both the period and amplitude of the oscillation of chromosome and inter-kinetochore distance increase with cell size in small cells, but saturate in large cells (Fig. 3). This has not been reported and still need further experimental verification. Without loss of generality, we can assume that *ξ*_*c*_ = *ξ*_*p*_ ≡ *ξ* in our calculation and discuss the phase diagram of chromosome movement (Fig. 4). Apparently, if the viscous friction is too strong, i.e., the dimensionless viscous drag *ξv*_0_/*Nf*_*s*_ is big, the system is overdamped and chromosomes cannot oscillate. For given viscous drag, chromosomes cannot oscillate too if the binding rate *k*_*b*,*i*_ is too small or the unbinding rate 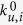 is too big (Fig. 4).

### Cell size affects the positioning of mitotic spindles in cortical pulling mechanism

**Figure 5:**
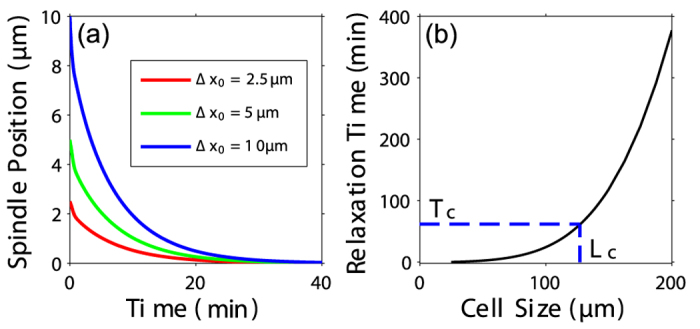
Cell size influences the positioning of spindles. (**a**) The positioning of spindle to the cell center for various perturbation Δ*x*_0_. (**b**) The relaxation time increases exponentially with cell size. 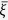 = 8 is used so that there is no chromosome oscillation.

We found that the spindle can be positioned to the cell center no matter whether the chromosome oscillates or not. For example, Fig. 5a shows when there is no chromosome oscillation, the spindle returns to the cell center after it is displaced from the center by Δ*x*_0_ and the time needed for the positioning process is roughly the same for various Δ*x*_0_ in a certain cell. If we use characteristic time *T*_1/2_ (the time at which the displacement of spindle Δ*x* decreases to Δ*x*_0_/2) to represent the relaxation time of the spindle, the relaxation time increases exponentially with cell size (Fig. 5b). This indicates that the spindle can be positioned to the cell center quickly in small cells, but in a cell bigger than a critical size *L*_*c*_, this cortical pulling and pushing mechanism can not center the spindle effectively since the relaxation time is even larger than the duration of metaphase *T*_*c*_. In very large cells, the astral microtubules do not reach the cortex, but the mitotic spindle can still be centered^47^,^48^. In this case, cells may use an alternative way, cytoplasmic pulling mechanism, to center the spindle^9^,^47^,^48^.

### The upper limit of spindle size

**Figure 6:**
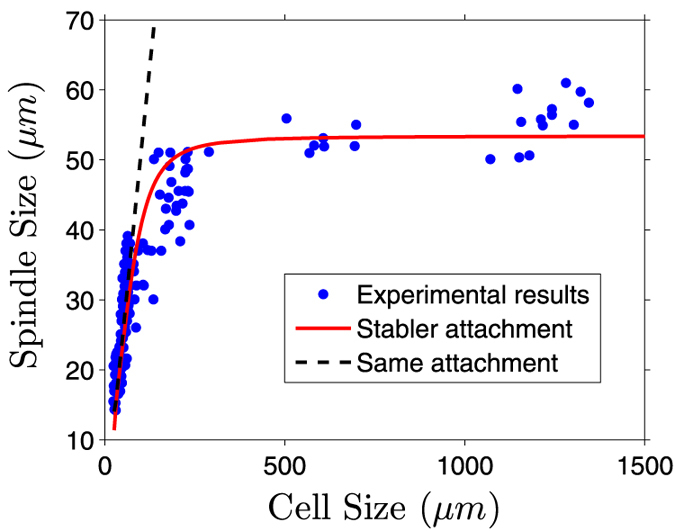
The property that the microtubule-kinetochore attachments are stabler than the microtubule-cortex attachments directly leads to an upper limit of spindle size. Our model can fit the experimental results^4^ perfectly if we assume 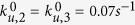 and 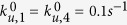 (red curve). In contrast, there is no upper limit for mitotic spindles if 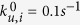 for *i* = 1,2,3 or 4 (black curve).

This model can also be used to study how spindle size is regulated by cell size. We found that the spindle size is always proportional to the cell size and there is no upper bound for the spindle size if we assume all the parameters are the same for *i* = 1,2,3 or 4 (black curve in Fig. 6). Notice that the microtubule-kinetochore attachments can be directly stabilized by tension^49^ or indirectly stabilized by suppressing the destabilizing activity of Aurora B^50^. Therefore, we assume that the microtubule-kinetochore attachments are stabler than the microtubule-cortex attachments. There are three ways to achieve this in the model: (1) 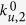 and 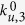 are smaller than 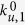 and 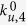; (2) 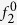 and 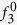 are smaller than 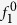 and 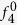; (3) both (1) and (2) are true. For all the above possibilities, we found that the size of mitotic spindles increases approximately linear with the cell size in small cells, but approaches an upper limit in large cells, which perfectly agrees with the experimental data during the early embryogenesis of Xenopus laevi eggs^4^. For example, we can fit the experimental results accurately if we only assume 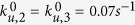 and 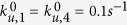 and other parameters are the same (red curve in Fig. 6). The stabler attachment between microtubules and kinetochores breaks the symmetry between kMTs and aMTs. The asymmetry is more obvious in large cells since the pushing force is position-dependent or length-dependent according to equation (5) in our model. Therefore, the spindle size does not scale with cell size any more. Although the spindle size can be tuned by factors, such as the number of building blocks^5^,^6^, the morphogen gradient^7^, or the growth velocity of microtubules^8^, our result indicates the intrinsic property of the spindle that the microtubule-kinetochore attachments are stabler than the microtubule-cortex attachments^49^,^50^ can directly lead to an upper limit of spindle size.

### The influence of the dynamic instability of microtubules

**Figure 7:**
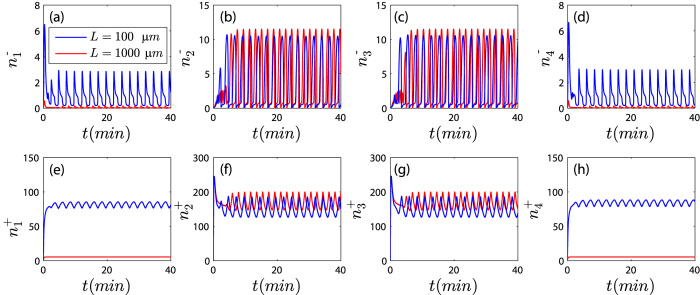
The populations of aMTs and kMTs during the chromosome oscillation when we assume *ρ*_*i*_ = *A*_*i*_/*l*_*i*_, where *A*_*i*_ = 300 *μm*/*s*. The populations of pushing and pulling astral microtubules (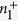, 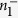, 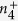 and 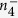) tends to vanish as cells become larger. Blue and reed curves indicate different cell size (*L* = 100 *μm* and *L* = 1000 *μm*).

In our model, *ρ*_*i*_ denotes the number of microtubules reaching the destination (cell cortex or chromosome) per unit time. Apparently, the growth and the dynamic instability of microtubules should play an important role on the value of *ρ*_*i*_. Experiments and theories have showed that there is an average growth rate which controls the transition between bounded growth and unbounded growth of microtubules^51^,^52^. The average behavior of microtubules in the two states is quite different. In the unbounded growth, microtubules switch between growing and shrinking states, but on average they have a constant growth velocity 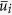. The length distribution propagates linearly with time and its shape evolves towards a Gaussian distribution^51^. If there is a wall at distance *l* away from the nucleation cite, it will take an average time 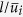 for microtubules to reach the wall. After that, the transition state is over, the peak of the microtubule length distribution will stop before *l*, and the length distribution reaches a steady state due to the existence of the wall. Therefore, in this case, we may simply assume *ρ*_*i*_ is a constant. In the bounded growth, microtubules switch between growing and shrinking states, but each microtubule will eventually shrink to the nucleating site at some time. Their average length saturates at some constant value due to the constant re-nucleation, and the length distribution at the steady state is a simple exponential distribution^51^. Apparently, the bigger distance the microtubule has to reach, the more unlikely microtubules will reach the destination (cell cortex or chromosome) since the length distribution at the steady state is an exponential distribution. Therefore, *ρ*_*i*_ should be the function of the distance *l*_*i*_ between the spindle pole and the destination. In our simulation, we found if we assume *ρ*_*i*_ = *A*_*i*_/*l*_*i*_ where *A*_*i*_ is a constant, the populations of pushing and pulling aMTs (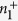, 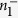, 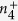 and 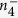) tends to vanish as cells become larger(see Fig. 7a,d,e,h). But kMTs still remain finite values. This is consistent with the experimental observations that aMTs are too short to reach the cortex in extremely large cells^47^,^48^. Notice that *ρ*_*i*_ also becomes a length-dependent parameter if we assume *ρ*_*i*_ = *A*_*i*_/*l*_*i*_. However, we found even we assume *ρ*_*i*_ is a constant, the upper limit of mitotic spindles still exists as long as the microtubule-kinetochore attachments is stabler. Therefore, *ρ*_*i*_ is not a dominant parameter regulating the spindle size. Except for the difference of microtubule population, we found a length-dependent *ρ*_*i*_ only changes our results quantitatively.

## Discussion

In this paper, we use a minimal model to identify the most essential factors that govern the oscillation, positioning and size limit of mitotic spindles. We show that mechanical force could be an effective tool to sense and regulate the size of subcellular structures, as has already been demonstrated in the growth and morphology of bacterial cells^53^,^54^,^55^. We found simple analysis, such as position-dependent forces generated by buckled microtubules and the intrinsic property that microtubule-kinetochore attachments are stabler than microtubule-cortex attachments, can explain the exiting data and provide some insightful predictions. Our model is based on a 1D cell, but it¡¯s possible to generalize it to two and three dimensions to study the position and orientation of the spindle for various cell shapes or when external forces are applied.

## Methods

The 12 coupled equations, i.e., equation (1)–(4, 22, 23, 24),(8) and (9), were solved by using MATLAB ordinary differential equation initial value problem solver ode15s. The initial values were taken to be approximately equal to the mean values of each unknown variable. The parameter values used in the simulation and the range of the parameter values tested in the simulation are given in Table 1. We can define three dimensionless parameters as 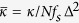, 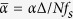 and 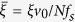. Based on the parameters listed in Table 1, we use the following values 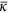 = 0.3, 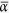 = 1 and 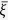 = 6 unless otherwise stated. The poleward flux is about 0.04 *μm*/*s*^59^,^60^. In our simulation, we found the value of this poleward flux has little influence on the size effects of the mitotic spindles. Therefore, without loss of generality, we assume *v*_*f*_ = 0 in the simulation. The cohesin complex connecting the two sister chromatids is simplified to be a linear spring. In the simulation, however, the force acting on the cohesin complex could be compressive and the two sister chromatids could switch their positions (*r*_3_ < *r*_2_) if the compressive force is too big. To avoid this situation, we use *α* = *α*_0_(1 + *δ*/(*r*_3_ − *r*_2_)^2*m*+1^), where *m* is an integer, *α*_0_ is a constant, and *δ* « 1. Therefore, *α* ≈ *α*_0_ when *r*_3_ − *r*_2_ is big, and *α* becomes very large when *r*_3_ − *r*_2_ is very small.

## Additional Information

**How to cite this article**: Jiang, H. Cell Size Modulates Oscillation, Positioning and Length of Mitotic Spindles. *Sci. Rep.* **5**, 10504; doi: 10.1038/srep10504 (2015).

## Figures and Tables

**Table 1:** Summary of the parameters used in the model.

**Parameter**	**Description**	**Value used in figures**	**Value tested in model**	**Reference**
*κ*	Bending rigidity of microtubules	21 *pN* ⋅ *μm*^2^	21 *pN* ⋅ *μm*^2^	[56]
Δ	Rest length of the cohesin bonds	1 *μm*	0.5 ∼ 2 *μm*	[44]
*α*	Spring constant of cohesin bonds	70 *pN*/*μm*	1 ∼ 100 *pN*/*μm*	[23,25]
*f*_*d*,*s*_	Stall force of dyneins	7 *pN*	7 *pN*	[39,40,57]
*v*_*d*,0_	Unloaded velocity of dyneins	0.2 *μm*/*s*	0.02 ∼ 2 *μm*/*s*	[39,40,57]
*F*_*s*_	Stall force of microtubules	4.2 *pN*	1 ∼ 10 *pN*	[58]
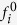	Characteristic force in the unbinding rate	*f*_*s*_/3	*f*_*s*_/10∼3*f*_*s*_	[57]
*ξ*_*c*_	Viscous drag coefficient of chromatids	2.1 *nN* ⋅ *s*/*μm*	0.1 ∼ 10 *nN* ⋅ *s*/*μm*	estimate
*ξ*_*p*_	Viscous drag coefficient of spindle poles	*ξ*_*c*_	0.1 ∼ 10 *nN* ⋅ *s*/*μm*	estimate
*N*	Number of motors on each microtubule	10	5 ∼ 15	[25]
*ρ*_*i*_	Number of MTs nucleated per unit time	15 *s*^−1^	0.5 ∼ 50 *s*^−1^	estimate
*k*_*c*,*i*_	Catastrophe rate of pushing microtubules	0.1 *s*^−1^	0.005 ∼ 0.2 *s*^−1^	[23,25]
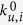	Unloaded unbinding rate of motors	0.07 or 0.1 *s*^−1^	0.005 ∼ 0.2 *s*^−1^	[19,20,57]
*k*_*b*,*i*_	Binding rate of motors	0.015 *s*^−1^	0.005 ∼ 0.2 *s*^−1^	[19,20,23,57]
